# A Return-to-Work Prognostic Model for Orthopaedic Trauma Patients (WORRK) Updated for Use at 3, 12 and 24 Months

**DOI:** 10.1007/s10926-016-9688-4

**Published:** 2016-12-23

**Authors:** Chantal Plomb-Holmes, François Lüthi, Philippe Vuistiner, Bertrand Leger, Roger Hilfiker

**Affiliations:** 1 Department for Musculoskeletal Rehabilitation, Clinique romande de réadaptation suvacare, Sion, Switzerland; 2 Institut de Recherche en Réadaptation, Clinique romande de réadaptation suvacare, Sion, Switzerland; 3 Département de l’Appareil Locomoteur, Hôpital Orthopédique, Lausanne University Hospital, Lausanne, Switzerland; 4 Institute of Social and Preventive Medicine, Lausanne University Hospital, Lausanne, Switzerland; 5 School of Health Sciences, University of Applied Sciences and Arts Western Switzerland Valais (HES-SO Valais-Wallis), Sion, Switzerland

**Keywords:** Rehabilitation, Vocational, Decision support techniques, Return to Work

## Abstract

*Purpose* Updating the Wallis Occupational Rehabilitation Risk (WORRK) model formula, predicting non-return to work (nRTW) at different time points (3 and 12 months) than in the validation study (2 years). *Methods* Secondary analysis of two samples was carried out (following orthopaedic trauma), including work status, the first at 3 months (428 patients) and the second at 12 months (431 patients) after discharge from rehabilitation. We used calibration (agreement between predicted probabilities and observed frequencies) and discrimination (area under the receiver operating characteristics curve) to assess performance of the model after fitting it in the new sample, then calculated the probabilities of nRTW based on the coefficients from the 2-year prediction. Finally, the intercepts were updated for both 3- and 12-month prediction models (re-calibration was necessary for the adjustment of these probabilities) and performance re-evaluated. *Results* Patient characteristics were similar in all samples (mean age 43 in both groups; 86% male at 3 months, 84% male at 12 months). The proportion of nRTW at 3 months was 63.8% and 53.4% at 12 months (50.36% at 2 years). Performance of the original WORRK for both 3- and 12-month prediction showed an AUC of 0.73, while statistically significant miscalibration was found for both time points (p < 0.001). After the updating of the intercept, calibration was improved and did not show significant miscalibration (p = 0.458 and 0.341). The AUC stayed at 0.73. *Conclusion* The WORRK model was successfully adapted by changing the intercept for 3- and 12-month prediction of nRTW, now available for use in clinical practice.

## Introduction

Work related and non-work related orthopaedic trauma constitutes a very important economic and social burden. In Switzerland alone, a country with 8 million inhabitants, the expenditure on direct costs, which involve all acute medical care and hospitalisation, rehabilitation and additional health care management, attains 1.23 billion US$, and indirect costs amount to an astounding 1.81 billion US$ (loss of earning and productivity as well as medical and worker compensation) [1–5]. On top of the financial load, orthopaedic trauma also leads to substantial disability and psychosocial strain, affecting quality of life, causing chronic pain and leading to prolonged inability to work, a factor which can have a negative effect on health (physical and psychological) as well as social integration [1, 3, 4, 6]. At first, as research was focused on major injuries, these effects were thought to be primarily as a consequence of life-threatening traumas. However, follow-up studies using the Abbreviated Injury Score (AIS) [7] scale to examine the efficacy of trauma centres made it clear that moderate and minor traumas contributed significantly or even more so than major traumas to the health burden [8].

It is now known that work is beneficial to health, and that return to work (RTW) can be used as an indicator of post-injury functioning and therefore the success of not only the acute-phase medical management but also long-term medical care such as rehabilitation and vocational programmes [1, 9, 10]. When considering what factors predict return to work, research shows that injury severity and medical factors alone cannot, especially as time passes and the injury becomes chronic. RTW prediction models for routine cases of low back pain (LBP) have supported the importance of these non-medical factors [11–14]. Other factors involved in predicting RTW are therefore being explored, with importance now being given to bio-psychosocial determinants such as job-related and socio-economic factors, patients’ psychological state and compensation and/or legal involvement; these factors are essentially the same when comparing LBP and orthopaedic trauma patients [1, 3, 10, 13, 15–19]. By recognising these variables, measurable early in recovery as they are mostly independent of the injury itself, it may be possible to adjust clinical decision making once out of the acute phase, with regards to physical and vocational rehabilitation programmes as well as compensation, in order to better distribute the resources available [1, 3].

In order to most accurately predict RTW status, it is important to identify an objective and reproducible screening method, applicable to a wide range of injuries and patients, including those with poor health literacy or language fluency. A model has already been developed and externally temporally validated by Luthi et al. [4], which applied at admission to rehabilitation, predicts non-return to work status at 2 years post-rehabilitation: the Wallis Occupational Rehabilitation Risk (WORRK) model (the formula is accessible by following the link beside the reference). This model includes 1 occupational, 6 biomedical and 12 psychosocial factors, and can be applied after orthopaedic trauma and for LBP patients; its difference and advantage over existing LBP prognostic models however is that it does not discriminate against non-native speakers, who make up a large proportion of the target population, therefore being applicable in patients that would otherwise be excluded because of this factor. Having access to this tool for prediction of work status at 3 and 12 months post-rehabilitation, however, could assist in decision-making earlier on in the rehabilitation process. The purpose of this study was therefore to externally temporally validate the already existing WORRK model, applied at admission to a rehabilitation centre, for 3- and 12-month prediction of non-return to work post-rehabilitation, after moderate and minor orthopaedic injury, allowing patients to be more rapidly screened and put into programmes best suited to their likely return to work outcome.

## Methods

The study took place at the “Clinique Romande de Réadaptation” (CRR), a Swiss accident insurance fund (SUVA—the main injury insurance in Switzerland) medical centre, where patients are sent on average 9 months after mostly traffic and work accidents if they exhibit persistent pain and functional limitations. Multidisciplinary therapeutic programs are put in place in order to improve functional status, quality of life, and the chance of returning to work. Using existing data from previous patient cohorts drawn from the CRR, we included patients with acute orthopaedic injuries (including all musculoskeletal localisations and AIS classifications [7]), admitted on average 9 months following the initial injury, and with information concerning their 3- and 12-month work status after discharge from the rehabilitation centre (representing their work status at, on average, 12 and 21 months following the initial injury), as well as information necessary for the predictors included in the WORRK prediction tool. We included patients that had no severe traumatic brain injury at time of accident (Glasgow coma Scale > 8), had no spinal cord injury, were capable of judgment, were not under legal custody and were not older than 62 years of age at the moment of hospitalization (to omit those who might opt for retirement rather than to RTW). Most of the patients were blue collar workers and were injured after traffic, work or leisure accidents [20, 21].

### The Swiss Insurance Framework

Health and accident insurances are compulsory in Switzerland; health insurance is financed by the individual, whereas each worker is insured against occupational and non-occupational accidents (as well as their consequences) by his/her employer and financed by monthly salary deductions. All construction and manual workers (i.e. blue collar workers) are insured by the Swiss National Accident Insurance Fund (Suva), which is the main accident insurer in the country. The accident and occupational disease insurances are in charge of providing daily financial allowances until there is a possibility of returning to work or until a disability pension is allocated. Disability insurance has set up specific structures to analyse the state of health and residual occupational capacity of the impaired workers. State of health is determined by a general practitioner and, if in doubt, by an acknowledged expert whereas vocational evaluation and rehabilitation are mainly carried out by specialised clinics [22].

The accident insurer must pay for medical treatment as long as a significant improvement in the state of health can be anticipated, without limit in terms of time or cost. The insured persons have a legal right to integration measures, but they are obliged to cooperate and do everything possible to return to an occupational activity, avoiding the need for pension allocation. If this is impossible, the disability insurance will help the worker in finding work, or look into the possibility of occupational reclassification and permit the insured person to obtain new occupational qualifications. With the intercession of the insurance institutions at an early stage in the form of vocational rehabilitation measures, the chances of work resumption and long-term reintegration are considerably increased, but if these measures fail, the disability insurance will have to pay a disability pension. Thus, reintegration measures are in the interests of the individual having had the accident, but also in the financial interests of the insurance company itself [23, 24].

### Transportability of the Published WORRK Model to Different Follow-up Intervals; Model Performance

We wanted to evaluate whether the WORRK prediction formula, which was developed for the prediction of non-return to work at 24 months after discharge from rehabilitation, could be used to predict non-return to work at 3 or 12 months in the same setting and with similar patients as used in the validation study of the original WORRK prediction model. These time points were chosen close to the end of rehabilitation treatment (3 months) and at 1 year because it is know that there is a steady increase in RTW in these patients during the first year, with then a plateau after 2 years, making this period potentially the most important in the recovery process [24]. To assess this, we evaluated the model performance of the published WORRK prediction tool with indices for discrimination and calibration. For discrimination, we calculated the area under the receiver operating characteristic (ROC) curve, as well as sensitivity, specificity, and positive and negative predictive values. For testing the calibration we used the Hosmer–Lemeshow test [25] and plotted the observed proportions of non-return to work against the predicted probabilities for groups defined by ranges (10%) of predicted risk as well as the slopes and calibration intercepts [26]. The calibration intercept is called calibration in the large and is calculated with a logistic regression with the slope fixed at one. If the coefficient is negative, the model will overestimate the probability of non-return to work; if the coefficient is positive, the model will underestimate the probability of non-return to work. Because the prevalence of non-return to work at 3 and 12 months is higher than at 24 months, we expected that this coefficient would be greater than zero. Because this sample comes from the same population as the samples for the development and validation of the original WORRK formula, we expected that the model would only need an update of the intercept, without revision of the model itself.

### Updating of the Prediction Model

Because the prevalence of non-return to work is different at 24, 12 and 3 months, we decided to update the intercept, as proposed by Steyerberg et al. [27]. After analysis of the calibration plot, we updated the intercept of the model for 3- and 12-month prediction separately. For this we fitted a logistic regression model in the new samples with the intercept as the only free parameter and using the linear predictor based on the previously published coefficients of the predictors as an offset variable (i.e. fixing the slope at unity). We did not update the prediction coefficients.

With the two new prediction formulae with the updated intercepts for the 3-month and 12-month follow-up, we re-evaluated the model performance (i.e. discrimination and calibration).

All analyses were done with Stata version 13.0 (College Station, Texas 77845 USA) and with R statistical software version 2.15.3 [28] with the packages PresenceAbsence (version 1.1.9).

## Results

From the different cohort studies, we included 428 patients with a 3-month follow-up and 431 patients with a 12-month follow-up. When analysing the overlap of the samples, 94 patients (17.9%) with a 3-month follow-up did not have a 12-month follow-up and 97 (18.5%) with a 12 month follow-up did not have a 3 month follow-up. The basic characteristics are quite similar for both follow-up time points (see Table 1). The non-return to work rate was, as expected, higher at 3 months (64%) than at the 12-month follow-up (53%).

**Table 1:** Characteristics of the patients for both follow-up time points

Variable	3 months, n = 428	12 months, n = 431	Difference between 3-month and 12-month samples (95% CI); p value
Men, n (%)	368 (86%)	364 (84%)	1.5% (−3.2 to 6.3%); p = 0.529
Age, mean (sd)	43 (10.9)	43 (10.5)	
Native speakers—French (%)	287 (67%)	270 (63%)	4.4% (−2.0 to 10.8%); p = 0.1758
Higher education (%)	268 (63%)	255 (59%)	3.5% (−3.1 to 10.0%); p = 0.300
Not returned to work at follow-up, n (%)	273 (64%)	229 (53%)	10.7% (4.1 to 17.2%); p = 0.002
Location: lower limb (%)	183 (43%)	182 (42%)	0.5% (−6.1 to 7.1%)
Location: back (%)	89 (21%)	99 (23%)	−2.2% (−7.7 to 3.3%)
Location: upper limb (%)	134 (31%)	132 (31%)	0.6% (−5.5 to 6.9%)
Location: multiple injuries (%)	22 (5%)	18 (4%)	1.0% (−1.9 to 3.8%)

*CI* confidence interval

### Model Performance for 3- and 12-Month Prediction Using the Formula Developed for Prediction at the 2-Year Follow-up

**Fig. 1:**
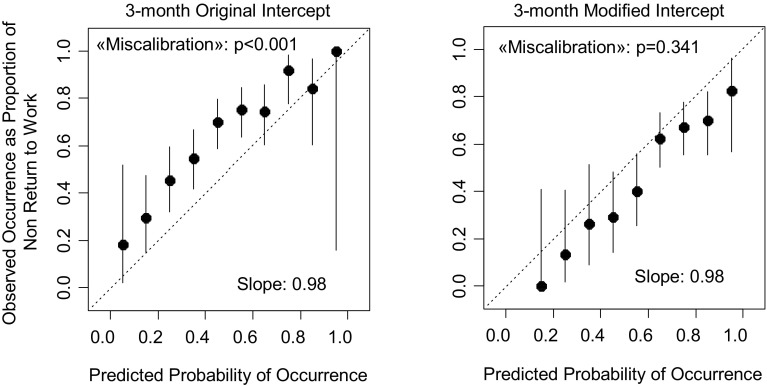
Calibration plots for the 3-month prediction with the original (*left*) and modified intercept (*right*)

**Fig. 2:**
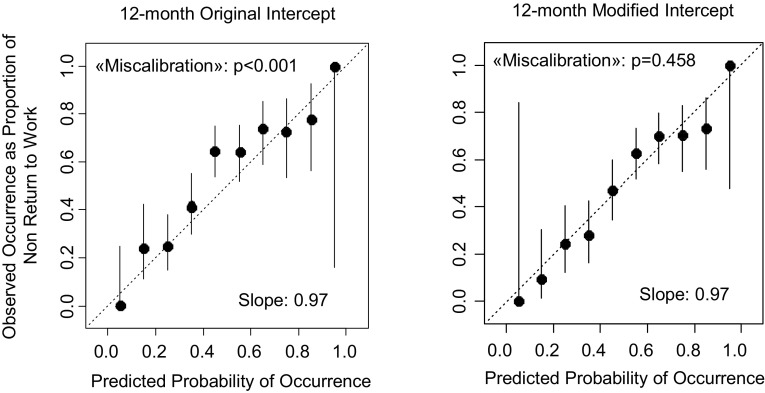
Calibration plots for the 12-month prediction with the original (*left*) and modified intercept (*right*)

The calibration plot showed that there was significant miscalibration for both the 3-month (p < 0.001) (Fig. 1) and 12-month (p < 0.001) (Fig. 2) prediction.

**Fig. 3:**
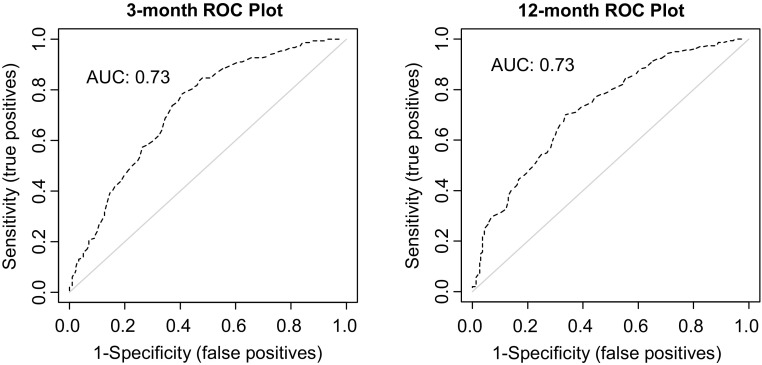
Receiver operating characteristic curves for the 3-month (*left*) and the 12-month prediction (only modified intercept shown). *AUC* area under the curve. *N* total number of participants with complete data for the variables in the model

The discrimination for 3- and 12-month prediction of non-return to work was moderate with an AUC of 0.73, which is equal to the published 2-year prediction [4]. See Fig. 3.

### Model Performance for 3- and 12-Month Prediction After Adaptation of the Intercepts

The original intercept as published by Luthi et al. in 2014 was −2.649848. This was adapted to −1.7850574 for the 3-month analysis, and to −2.978829 for the 12-month analysis.

After the modification of the intercepts, the calibration was better and with a non-significant test for deviation from perfect calibration (p = 0.341 for the 3-month prediction and p = 0.458 for the 12-month prediction). See Figs. 1 and 2.

The discrimination remained the same with an AUC of 0.73.

The sensitivity, specificity, as well as the positive and negative predictive values for different cut-off points of the predicted probability of non-return to work based on the adapted prediction formulas are presented in Table 2. For example, if a threshold of 0.5 is used, of 100 patients predicted to not return to work, at 3 months 28 will have returned to work (PPV 72) while at 12 months 31 will have returned to work (PPV 69).

**Table 2:** Sensitivity (SN), specificity (SP), as well as the positive (PPV) and negative (NPV) predictive values for different cut-off points of the predicted probability of non-return to work based on the adapted prediction formulas

Threshold	3-Month prediction non-return to work						12-Month prediction non-return to work					
	n predicted positive	SN	SP	PPV	NPV	% c.cl	n predicted positive	SN	SP	PPV	NPV	% c.cl
≥0.1	428	100	0	64	0	64	429	100	1	53	100	54
≥0.2	421	100	5	65	100	65	408	99	10	56	91	58
≥0.3	403	97	12	66	72	66	367	95	26	59	81	62
≥0.4	376	95	25	69	75	70	317	89	44	64	77	68
≥0.5	333	88	39	72	64	70	251	75	61	69	68	68
≥0.6	268	74	58	76	56	68	165	52	77	72	58	63
≥0.7	181	53	76	80	48	61	89	28	88	73	52	56
≥0.8	92	30	93	88	43	53	42	14	95	76	49	52
≥0.9	22	7	97	82	37	39	5	2	100	100	47	48

*c.cl* correct classified

## Discussion

In this evaluation of the Wallis occupational rehabilitation risk (WORRK) model, applied to a cohort of patients at admission to an occupational rehabilitation programme following minor or moderate orthopaedic trauma, it can be concluded that the WORRK model, originally built for the prediction of non-return to work status at 2 years post rehabilitation, can be used for the prediction of 3- and 12-month work status, by changing simply the intercept of the model, as the baseline risk for non-return to work is not the same at 3 and 12 months in comparison to 2 years.

The effect of rehabilitation on chronic low back pain is known to influence very little RTW [29]. Though the rates of nRTW after rehabilitation found in our acute orthopaedic trauma patients may seem low, they fall within the rates found in similar populations (Clay et al. [1] report rates ranging between 15 and 58%).

The strength of this study is the systematic approach, the large sample size and the application of the model at two different follow-up time points. The strengths of the WORRK model are first of all that it is one of the only systematic tools that is an improvement on an existing model, having used recalibration in order to apply it to different follow-up time points [30]. This is advantageous over other models that are validated at a certain follow-up time point, and then arbitrarily applied to different time points without recalibration. Additionally, it allows the inclusion of patients with poor health literacy or language fluency. Incorporating this population into the analysis is important as they are an increasing presence in the work force of industrialised countries, are at risk of adverse work conditions, and may have cultural expectations or representations hindering return to work [4, 8, 31–34]. Moreover, the WORRK model includes twelve psychosocial factors (including language, education and profession, but also social vulnerability, mental health threat and coping) making it applicable in a wide range of socio-economic environments.

The three main limitations are first that the calibration at 3 months is slightly inaccurate and the model might benefit from a recalculation of the coefficients or the addition of new predictors. However, this would need a larger sample size, and it was therefore decided not to carry out this recalculation. The second limitation is that this study only provides a temporal external validation [35]; in order to be able to recommend the WORRK model in other settings and health systems (for example where compensation bodies are not available), an external validation (applicable in other centres) is necessary. Thirdly, it must be noted that certain important notions with regards to RTW such as self-efficacy and information about the workplace environment, are not measured by the model, as they were not available in a standardised manner at the time [36]. A revision of the model should address this issue.

When comparing this model to other available prediction models, there are, to the best of our knowledge, no other prediction tools that are clinician rated. However, there are prediction models using a workers compensation-claims database [3], performance-based measures [37], performance-based measures combined with self-reported ability [38] and purely self-reported questionnaires via the OMPSQ (Orebro Musculoskeletal Pain Questionnaire) [39]. These models may be difficult to apply in an acute rehabilitation setting for the following reasons, respectively: where compensation bodies may not yet be involved or not available at all (for example as is the case in the UK), where performance may still be suboptimal due to injury, and in a chronic rehabilitation setting where self-reported ability can be biased by long-term sick leave as well as poor health literacy or language fluency [31]. Moreover, although using purely insurance-based data provides excellent prediction, this type of model is not pertinent in different socio-economic or insurance settings. The WORRK model is therefore an innovative applicable tool for the acute and chronic rehabilitation setting, providing an objective and accessible prediction of work status at 3, 12 and 24 months.

Moreover, the WORRK model might be useful in clinical practice with regards to the decision making process. For example, in situations where the duration and program of the rehabilitation depends on the prognosis, our model might inform clinicians earlier in the chronology of the patient. This could be particularly useful as 1 year post-rehabilitation seems to potentially be the most important period in the recovery process [24]. These decisions may also improve the efficient allocation of scarce resources. However, the effectiveness of the application of this tool is still to be evaluated in a randomized controlled trial, a study which is currently underway (NCT02396173).

With regards to use in research, the results of this study suggest that the WORRK model can be used, with a modified intercept, for the prediction of shorter follow-up time points. This is important, for example, in randomized controlled trials for the inclusion or stratification of patients, as well as in observational studies where it is important to control for confounding [40]. With our update of the intercepts at 3 and 12 months, this is now possible for studies with follow-up time points of 3, 12, or 24 months.

In conclusion, the Wallis Occupational Rehabilitation Risk Model (WORRK), which until now has been validated for the prediction of work status at 2 years post rehabilitation following minor or moderate orthopaedic trauma, has now been temporally externally validated for the prediction of work status at 3 and 12 months by changing the intercept of the model. Use of this model in clinical and research settings may then be used to screen patients, particularly at 12 months, assisting in decision-making and allocation of appropriate rehabilitation programmes and funds.
